# Contributions of biogenic material to the atmospheric ice-nucleating particle population in North Western Europe

**DOI:** 10.1038/s41598-018-31981-7

**Published:** 2018-09-14

**Authors:** D. O’Sullivan, M. P. Adams, M. D. Tarn, A. D. Harrison, J. Vergara-Temprado, G. C. E. Porter, M. A. Holden, A. Sanchez-Marroquin, F. Carotenuto, T. F. Whale, J. B. McQuaid, R. Walshaw, D. H. P. Hedges, I. T. Burke, Z. Cui, B. J. Murray

**Affiliations:** 1 Institute for Climate and Atmospheric Science, School of Earth and Environment, University of Leeds, Woodhouse Lane, Leeds, LS2 9JT UK; 2 School of Chemistry, University of Leeds, Woodhouse Lane, Leeds, LS2 9JT UK; 3 Institute of Biometeorology, National Research Council (IBIMET-CNR), Via Caproni 8, 50145 Florence, Italy; 4 School of Earth and Environment, University of Leeds, Woodhouse Lane, Leeds, LS2 9JT UK; 5 Earth Surface Science Institute, School of Earth and Environment, University of Leeds, Woodhouse Lane, Leeds, LS2 9JT UK; 6 Present Address: NHS Digital,1 Trevelyan Square, Boar Lane, Leeds, LS1 6AE UK; 7 Present Address: Institute for Atmospheric and Climate Science, ETH Zurich, Zurich, Switzerland

## Abstract

A minute fraction of atmospheric particles exert a disproportionate effect on the phase of mixed-phase clouds by acting as ice-nucleating particles (INPs). To understand the effects of these particles on weather and climate, both now and into the future, we must first develop a quantitative understanding of the major INP sources worldwide. Previous work has demonstrated that aerosols such as desert dusts are globally important INPs, but the role of biogenic INPs is unclear, with conflicting evidence for their importance. Here, we show that at a temperate site all INPs active above −18 °C at concentrations >0.1 L^−1^ are destroyed on heating, consistent with these INPs being of biological origin. Furthermore, we show that a global model of desert dust INPs dramatically underestimates the measured INP concentrations, but is consistent with the thermally-stable component. Notably, the heat sensitive INPs are active at temperatures where shallow cloud layers in Northern Europe are frequently observed to glaciate. Hence, we suggest that biogenic material is important for primary ice production in this region. The prevalence of heat sensitive, most likely biogenic, INPs in this region highlights that, as a community, we need to quantify the sources and transport of these particles as well as determine their atmospheric abundance across the globe and at cloud altitudes.

## Introduction

The phase transition of water to ice in supercooled droplets can bring about precipitation and modulate the radiative properties of clouds^1,2^. Atmospheric aerosol can contain ice-nucleating particles (INPs)^3^, which permits primary ice nucleation at temperatures above about **−**33 °C, where the homogenous nucleation of supercooled water is so slow that water would otherwise persist in a supercooled state^4^. Common aerosol particles that are thought to be effective as INPs in the mixed-phase cloud regime include desert dusts^5–7^ and, in remote marine locations where dust concentrations are low, marine sea sprays^8,9^. While desert dust is thought to be a globally important INP type^5,6^, it has been found that shallow clouds over Morocco only glaciated below **−**18 °C, despite being collocated with dust^10^. In contrast, similar clouds in other terrestrial regions, such as N. Europe, typically glaciate at temperatures up to or even above **−**10 °C^11^. This difference is thought to be related to differences in INP populations rather than other variables such as cloud properties or meteorology^11^, but the INP type that drives this relatively high temperature nucleation in Northern Europe is unknown. While certain bioaerosols are known to be exceptional in their ice-nucleating abilities^7,12^ and have been posited as drivers of ice formation in clouds at low supercoolings^13–19^, there are conflicting conclusions regarding their global importance^20,21^. Although biogenic INPs have been examined since the 1960s (for extensive reviews of the general topic of ice nucleation, see Ashworth *et al*.^22^ Kanji *et al*.^23^ Hill *et al*.^24^ and Murray *et al*.^12^), a major source of uncertainty concerning the impacts of biogenic INPs is due to the lack of field studies that have determined their abundance relative to other ambient atmospheric INPs.

Biogenic components of INPs have previously been detected in precipitation samples collected at sites across the globe^25–28^. Estimates of atmospheric INP concentrations at cloud altitudes can be made based on the INP content of precipitation^29^ and heat tests suggest that some of these INP are biological^25,27^. In addition, measurements indicate that there is a major biogenic INP component in cloudwater at Puy De Dôme in France^28^. Also, Garcia *et al*.^30^ monitored the amounts of biogenic INPs released to the air from crops pre- and post- harvest in Colorado and Nebraska, and found using heat tests on sampled aerosol that a major proportion of emitted INPs were biogenic. In forested ecosystems, INPs have also been found to correlate well with the amount of fluorescent bioparticles and carbonaceous material in the aerosol^31,32^. Further suggestions for the importance of biological INPs in atmospheric ice nucleation have emerged from particle mass spectrometry of cloud ice crystal residues^16,33^. Notably, however, significant questions on the use of particle mass spectrometry to identify bioaerosols remain, in particular due to the potentially large misattribution rate based on the selected markers used^34^. Overall, despite there being evidence for biogenic INP in rain water and associated with specific events, such as harvests, there have been very few measurements of the relative importance of biological INPs versus other INP types in ambient aerosol samples around the world and none in the atmosphere of the UK.

Given the dearth of information on the contribution of biogenic INPs to the total burden, it is perhaps unsurprising that models simulating the impacts of biologically-derived INPs on clouds come to differing conclusions. In an aerosol-climate model, Hoose *et al*.^20^ simulated the global annual average INP concentrations using a subset of known biogenic ice-nucleating particles, including *P*. *syringae* bacteria. They found the proportion of nucleation due to bioaerosols was very small (≤0.6%). Similarly, Spracklen and Heald^35^ concluded that the fungal spores and bacteria only contribute a minor proportion of INPs, but do suggest that they may contribute to INP populations at warmer temperatures in some locations. However, these studies did not consider the possible roles of subcellular INPs, such as macromolecules, which are generated in large amounts by certain fungi and plants (e.g. pollen)^36–40^. These nanoscale entities could substantially increase the relative importance of biogenic particles as INPs^37,41^, especially if they become internally mixed with other aerosol particle types^42,43^. Conversely, using a cloud system resolving model, Phillips *et al*.^21^ concluded that cloud properties such as ice crystal number and precipitation are significantly influenced by bioaerosol particles when bioaerosol concentrations are elevated above average (but within previously observed levels). Despite these and other attempts to simulate the net contribution of biogenic INPs to cloud properties, the lack of field studies examining the biogenic INP burden in atmospheric aerosol makes it impossible to accurately predict their influence on clouds.

## Results and Discussion

**Figure 1:**
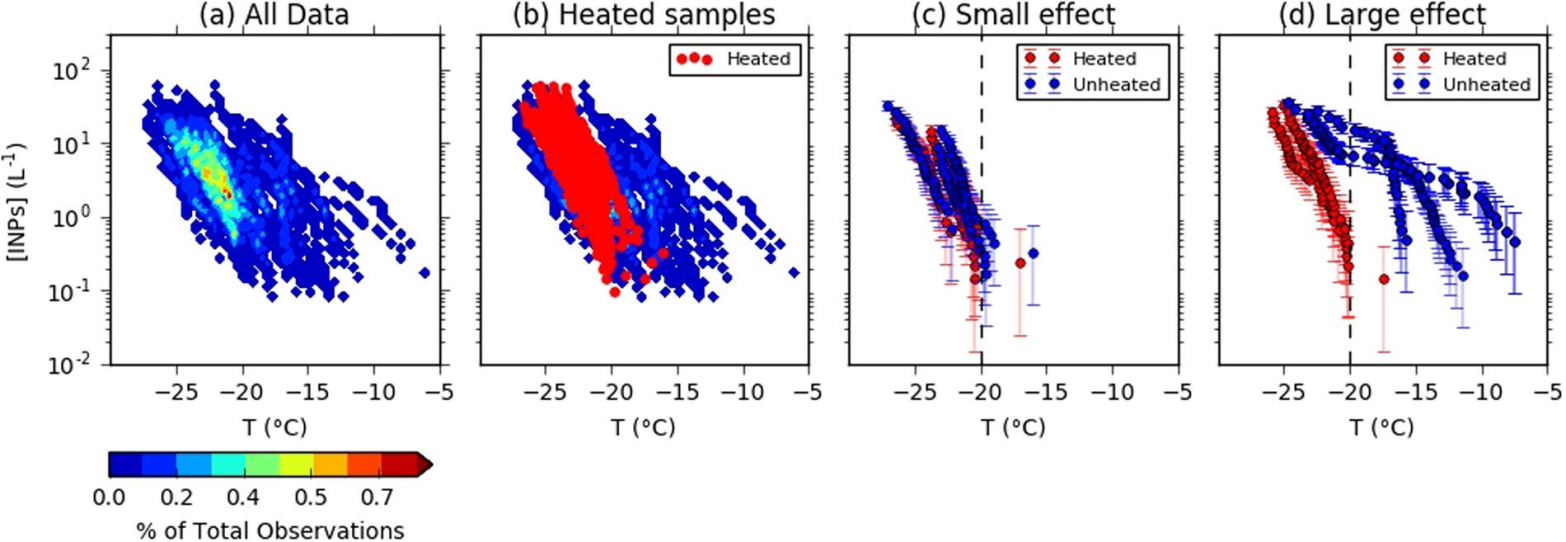
Results from the six week INP measurement campaign at a rural site in the UK. Illustrated in (**a**) are INP concentrations observed throughout the campaign, colour-coded to the frequency of observation. Results of the heat tests are shown in (**b**). Also shown are cases when heating had only a small effect (**c**) and a large effect (**d**) on the activities above −20 °C.

To quantify and characterise INPs in the atmosphere at a temperate site in Europe, a six week campaign was held at a rural location in the UK. INP concentrations at the site throughout the campaign are illustrated in Fig. 1a, where it can be seen that concentrations span more than two orders of magnitude at a particular temperature. For example, at an activation temperature of −18 °C, the INP concentration ranged from about 0.1 to 10 L^−1^. Such a wide variability has previously been seen at other ground based measurement sites. For instance, at a rural site in Italy, Belosi *et al*.^44^ report concentrations at −18 °C to fall within the range 0.07–1 INP L^−1^. Despite the wide variability in INP concentrations, Fig. 1a shows that there is a strong peak in frequency centred at about 2 INP L^−1^ at −22 °C. This distinct maximum in the INP concentration frequency plot is consistent with a single persistent INP type, whereas the concentrations at higher temperatures are consistent with a much more variable INP type.

Determining the nature of the INPs which make up the population in a typical atmospheric INP spectrum is challenging. This is in part because biogenic INPs are highly varied, being composed of whole cells, cell fragments or subcellular entities, such as proteins, which become internally mixed with other aerosol types, hence they are very difficult to identify directly. In this study we have used the commonly used heat treatment to assess the contribution of biogenic INPs to the overall INP population. This test is based on the fact that many known biological ice-nucleating materials nucleate ice because of the presence of specific ice-nucleating proteins. These proteins are disrupted and denatured on heating which inhibits their ice-nucleating ability whereas inorganic ice-nucleating materials tend to be insensitive to heat, hence heating a sample in water has become a standard and effective test for protein based biological ice-nucleating material^9,45^. Conversely, known inorganic INPs such as mineral dusts are not affected by heating to temperatures of about 100 °C^46,47^. Previously, studies have focused on examining the effects of heat treatment on either surface-collected samples such as soils^45,46,48^, leaf litter^14,49^ and sea water samples^9,50^, or on particles found in precipitation^27,51^ and aerosol collected during harvest periods^30^.

In the current study, greater than 100× reductions in INP concentrations were observed in the samples after heating to 95 °C for 1 hour, with reductions occurring mainly at *T* > −20 °C (Fig. 1b,c). In Fig. 1b, the heated samples are plotted alongside the original data. Most activity above −18 °C was removed on heating. At −20 °C, substantial reductions in activity (more than a factor of 2) were observed in 59% of heated samples. Conversely, for samples where nucleation was primarily observed to occur at temperatures below −20 °C, the effects of heating were less pronounced (Fig. 1c) than when nucleation began in the original samples above −20 °C (Fig. 1d). Following the loss of heat-labile INPs in the samples, the remaining activity was found to converge at the most frequently observed values prior to heating. This observation implies that there are at least two distinct populations of INPs in the samples with heat-labile INPs becoming more prominent at *T* > −20 °C, and a heat-resistant INP type at lower temperatures. It should be noted that heating does not eliminate all the biogenic INPs in a sample. A variety of biogenic INPs in surface soils are resistant to heat treatments below 100 °C^45,48^. Accordingly, the loss of INPs on heating can be viewed as a lower limit to the number of biogenic INPs in the sample. Overall, we find that the majority of INPs active at temperatures warmer than −18 °C at concentrations greater than 0.1 L^−1^ are destroyed on heating, and most likely of biological origin. Inspection of a filter with scanning electron microscopy revealed several biological particles which were most likely fungal spores (see SI Fig. 6). This shows that biological aerosol were present at this site, which is consistent with the presence of heat sensitive, most likely biological ice nucleating aerosol.

**Figure 2:**
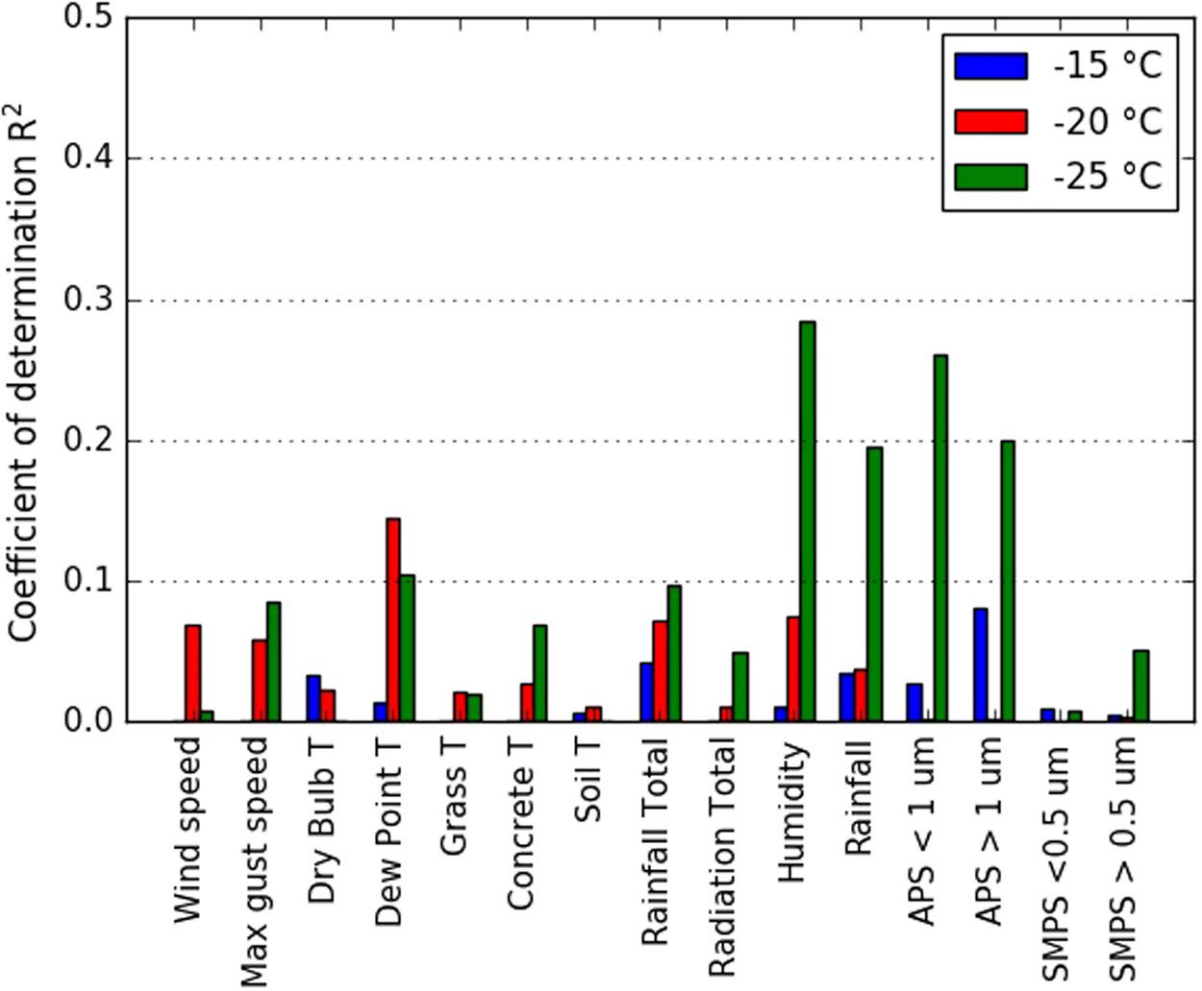
R^2^ correlation coefficients between log_10_[INP] and various meteorological parameters measured on-site. Illustrated are linear correlations to meteorological data collected from the collocated MET Office station, as well as total counts from the aerodynamic particle sizer (APS,) and scanning mobility particle sizer (SMPS). The duration of the sampling time was on average 3 hours (see SI Tables 1 and 2 for further details). The APS counts determined the number of particles with an aerodynamic diameter >0.5 µm, while the SMPS counted particles with mobility diameters in the range 14–710 nm. Poor linear correlation of INP concentrations with the indicated variables was observed.

**Figure 3:**
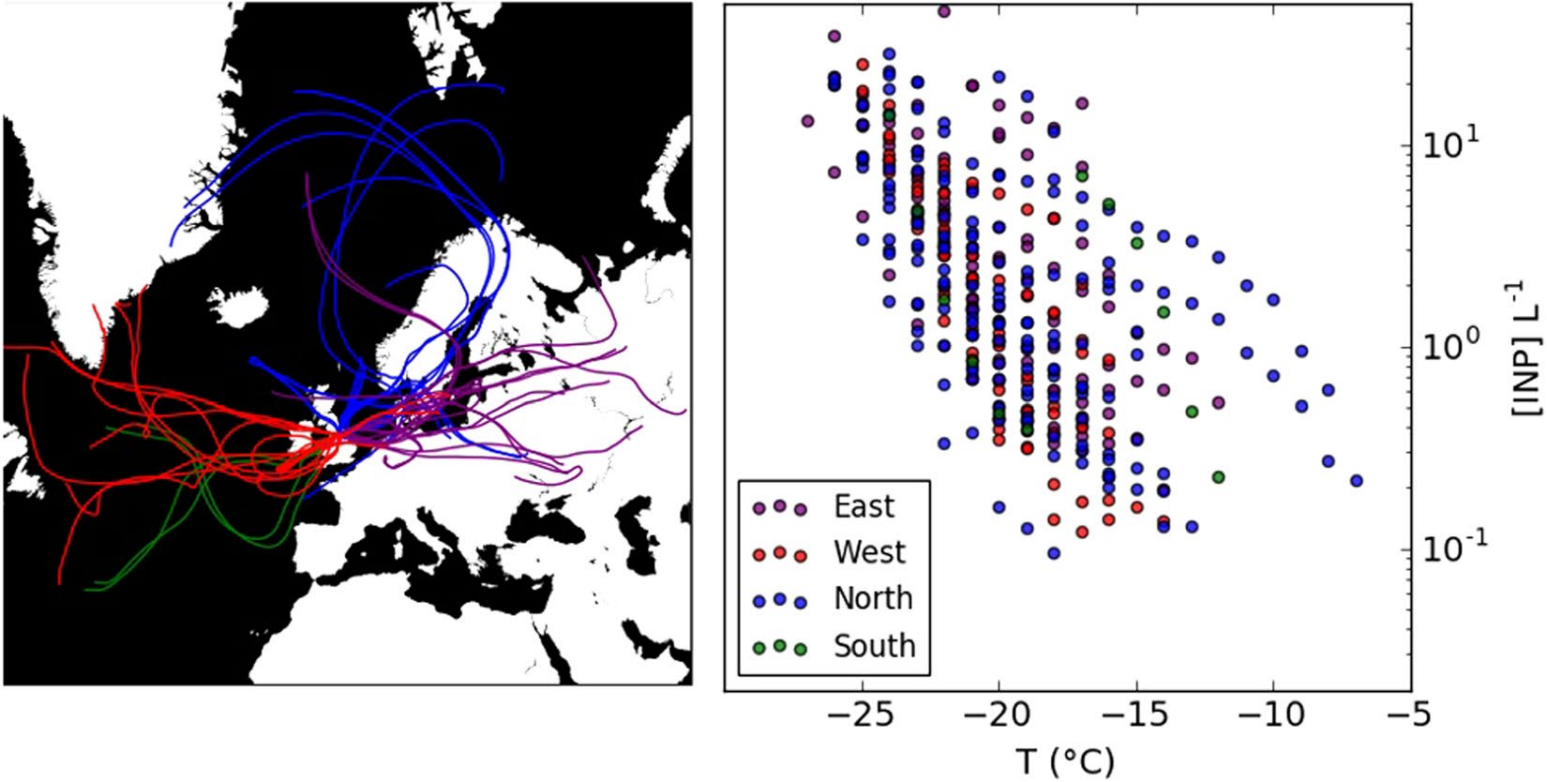
INP concentrations colour coded according to the origin of air mass, as taken from the major direction of the air mass during the 2 days prior to arrival at the site using the 5 day HYSPLIT v4 back trajectory calculator.

To investigate the sources of INPs, it was examined whether INP concentration was correlated with local meteorological variables including wind speed, ambient relative humidity and rainfall. The extent of correlations with local meteorological data is variable in literature data and changes from site to site^44,52^; although rainfall has been correlated with high INP concentrations in several locations^26,31,53^. For soil-derived particles, threshold friction velocities increase with factors such as soil wetness, which in turn is related to rainfall and surface relative humidity^54^. Conversely, increasing relative humidity has also been suggested to trigger the release of ice-nucleating aerosols from local biota^55^, while rain splashing on leaves and on soils^56,57^ has also been suggested to be a driver of biological INP emissions. In the current study, however, the coefficients of determination, *R*^2^, suggested little linear correlation between INP concentrations and a range of meteorological factors (Fig. 2). In addition, air mass back trajectories using the NOAA HYbrid Single Particle Lagrangian Integrated Trajectory (HYSPLIT) model^58^ did not indicate strong links between the origin of air masses and INP concentrations (Fig. 3). This is in contrast to what would be expected if there were a strong source of INPs within 2 days transport. For example, if the urban areas west of the sampling site were a strong source, we would anticipate a strong correlation with wind direction, or if there were a strong local source associated with rainfall then we would anticipate a correlation with rainfall. Instead, it seems that the INPs at this site are highly variable, but without an obvious local source. Given the landscape around the sampling site is representative of similarly heterogeneous landscape across North Western Europe and there is no dependence on air trajectory we suggest these results are representative of the wider region, although more measurements in more locations are needed to test this.

**Figure 4:**
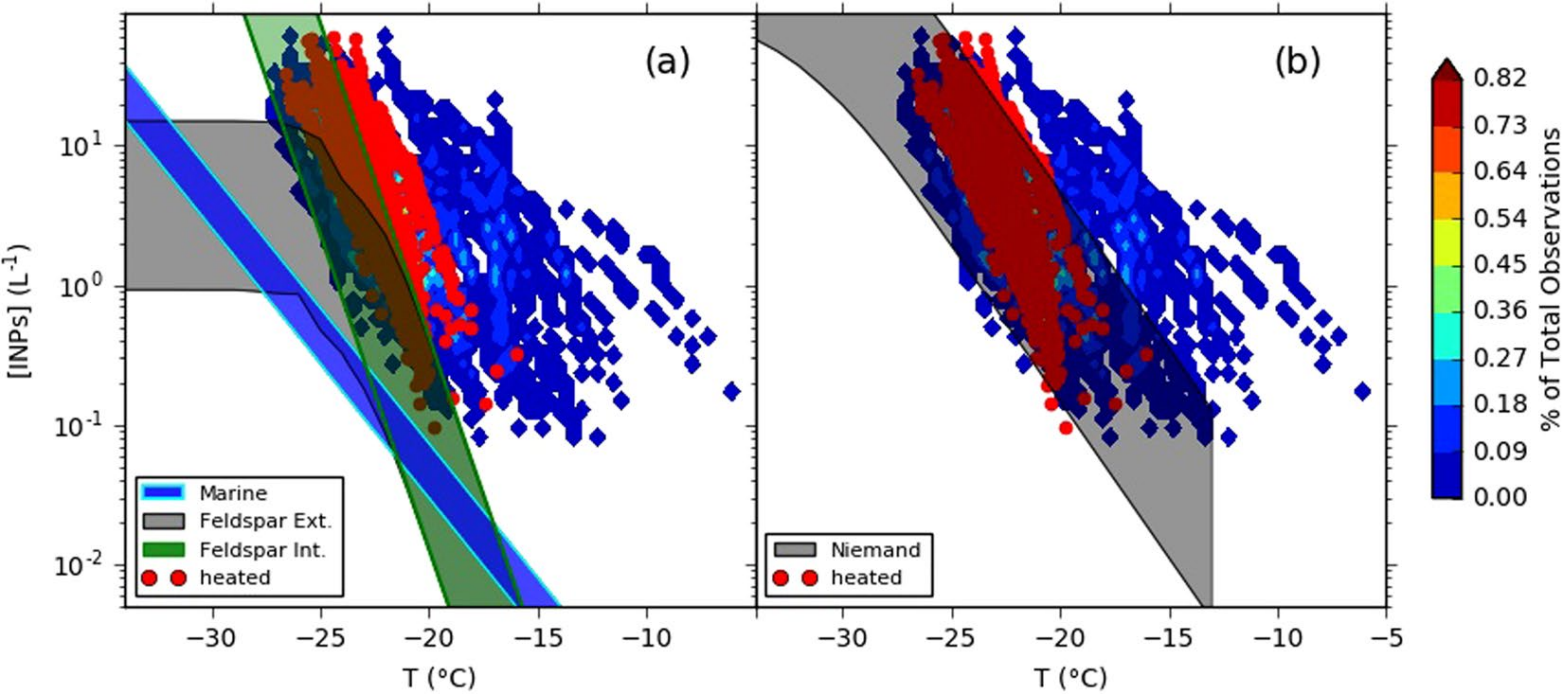
Comparison of measured INP concentrations to those predicted using GLOMAP as detailed by Vergara-Temprado *et al*.^6^ (**a**), which uses parameterisations for ice nucleation activities based on K-feldspar and marine biogenic INPs, and separately, using the N12^61^ parameterisation for ice nucleation from desert dust (**b**). Terrestrial biogenic INPs are not parameterised in the model, which we suggest accounts for the under-predictions of the simulation at warmer temperatures. The width of the model predictions are based on the 20^th^ and 80^th^ percentiles of the daily averaged model outputs. The colours of the untreated data represent the frequency of observation, as in Fig. 1a.

We now examine what the heat resistant INP type we observe may be. Desert dust is thought to be a globally important INP type^16,59^ with a lifetime in the accumulation mode on the order of days to weeks, hence it is transported in sufficient concentrations to contribute to the INP spectrum far from source regions^6^. Indeed, the fact that the back trajectories show that the sampled air did not recently pass over desert regions is entirely consistent with the presence of desert dust at our sampling location. To determine if the heat resistant INP we observed are consistent with transported desert dust, we compared our results with those predicated by the Global Model of Aerosol Processes (GLOMAP)^6^. INP concentrations simulated in GLOMAP from desert dust (K-feldspar) and marine sea spray emissions, i.e. neglecting other INP species such as terrestrial biogenics, were within one order of magnitude of 57% of the INP observations from around the globe^5,6^. As can be seen in Fig. 4a, it is expected that marine biogenics, whose nucleating activities are those parameterised by Wilson *et al*.^9^, are predicted to be of minor importance at this location. In Fig. 4a, we also show the concentration of INPs predicted from transported desert dusts, assuming the K-feldspar component is responsible for nucleation. K-feldspars have been demonstrated to show a far larger ice-nucleating activity than other minerals such as quartz, kaolinite or calcite^5^. Being the most ice-active mineral in desert dust found to date, and given its natural abundance, K-feldspar has been suggested to be of first order importance in determining the ice-nucleating activity of lofted desert dusts. Accordingly, the nucleating activities used here are taken from Atkinson *et al*. (A13)^5^ in conjunction with the modelled global K-feldspar distribution in order to estimate the INP concentration associated with desert dust. The initial fraction of desert dust which is feldspar is derived from soil composition maps from Claquin *et al*.^60^ Of this feldspar, 35% is assumed to be K-feldspar. Further details can be found in Vergara-Temprado *et al*.^6^. Two limiting assumptions are made, one where K-feldspar is externally mixed and one where it is internally mixed throughout the dust particle population. In reality, dust particles will be somewhere between these two mixing assumptions. In Fig. 4b, the desert dust activity was defined by the parameterisation of Niemand *et al*.^61^ (N12), which is based on surface samples from areas including the Nile delta, the Sahara and Israel.

Given the uncertainties in the accuracy of the model, due to uncertainties in the transport of dust and parameterisations of mineralogy and ice nucleating activities^6^, the agreement between the model-predicted INP concentrations and the heat resistant INPs is reasonable. Both the A13 and N12 parameterisations were found to yield reasonable agreement to observed heat-resistant INP concentrations. For the A13 parameterisation, the internal mixing assumption gives better agreement than the external mixing assumption. Overall, the thermally resistant component of INPs at this site is consistent with transported desert dust, defined by either N12 or A13, possibly together with heat resistant biological material. The presence of mineral dust within the aerosol at this site was confirmed through conducting energy dispersive x-ray analysis on a sample collected on the 17^th^ Oct (see SI Fig. 5). We record the presence of particle compositions consistent with aluminosilicates and silicates dominantly in the coarse mode, amongst other aerosol types. Whether this dust is derived from desert regions or is from local sources is unclear, but the presence of mineral dust is consistent with the thermally stable INPs we detect in our experiments and the results of our two-component global INP model.

It is also evident from Fig. 4 that the model dramatically under-predicts the total (non-heat treated INP population) on many days. We have to be cautious when comparing a global model result with large grid boxes (2.8° × 2.8°) to a measurement at a single location since local emissions may strongly influence the measurements, but may make a minor contribution to concentrations on a model grid box scale. However, the comparison is valid if the site is representative of the grid box. Given we observe no evidence for strong specific local emissions, we have argued above that our measurements are broadly representative of the region. Testing this assumption will require far more measurements over a longer temporal and larger spatial scale than the present measurements. Nevertheless, the comparisons indicate that the model is missing a heat sensitive, most likely biogenic, source of INP. This is consistent with other work. For example, comparison of the GLOMAP INP model with measurements from around the world, revealed that in many mid-latitude terrestrial locations the INP concentrations were higher than the model predicted at temperatures warmer than −15 °C^6^, similar to this study. Also, shallow clouds were observed to glaciate at temperatures up to or even above −10 °C in N. Europe^11^, which is hard to explain with desert dust only and may be consistent with an additional source of INP. Overall, we suggest that there is an important missing source of INPs in the model and based on the heat tests we suggest that this missing source is most likely biogenic.

## Conclusions

This work demonstrates that a major fraction of INPs found at a temperate rural site in Europe were sensitive to heat and therefore likely biogenic in origin, with this fraction becoming more important at warmer temperatures. Given that INPs active at several temperature intervals were found to be poorly correlated with local meteorology and back trajectories, the source of the heat-labile INPs remains unclear. Given that the landscape around the sampling site is similarly heterogeneous to much of the UK and Western Europe, it is argued that the results are of regional relevance. When a global model with no intrinsically defined terrestrial biogenic INP sources was used to examine if we could explain observed INPs above −20 °C, it under-predicts observations. This loss in the predictive capacity of the model coincides with temperatures where INPs were observed to be thermally-labile, suggesting an additional model source of heat labile, most likely biogenic, INPs is needed to account for the observations. This raises the possibility that the INP population in the atmosphere in this region is controlled by biogenic emissions from the terrestrial environment, which may be natural or linked to human activity. Accordingly, there is a possibility that INP populations, and their impact on clouds and climate, may be influenced by changes in land-use. Hence, work should continue to define the sources of INP in the mid-latitudes, determine their variability across a range of temporal scales and characterise their properties and transport.

## Methods

### Sample collection and analysis

Field experiments were conducted at the University of Leeds Research Farm (54 m above sea level; N53°52′7.086′′ W1°19′8.489′′) from 19/09/16 to 02/11/2016, during which time a mobile laboratory (the “IcePod”) equipped with a PM_10_ inlet (model URG-2000-30DBN-A, URG) and with an aerosol subsampling system was stationed at the site. Ambient particles were sampled onto 0.40 µm Nucleopore track-etched membrane polycarbonate filters at a flow rate of 5 L min^−1^. Note that these filters collect particles across the full aerosol size range, including at sizes below the size of the pore, with high efficiency^62,63^. Handling blanks, where the sampling system was set up and a HEPA filter placed on the inlet, were conducted to verify that in the absence of ambient particles contamination was not an issue (see SI). The average sampling time was approximately 3 hours, and the average volume of air sampled was 890 litres (see supplementary information). Further information can be found in Supplementary Information (SI) Tables 1 and 2. Aerosol number and size distributions were determined using both a Scanning Mobility Particle Sizer (SMPS) spectrometer (Model 3936, TSI), and an Aerodynamic Particle Sizer (APS) spectrometer (Model 3321, TSI). For comparison, electrical mobility diameters from the SMPS and aerodynamic diameters from the APS were converted to volume equivalent diameters as per Möhler *et al*.^64^. A compilation of all the particle size distributions is shown in SI Fig. 2, where the contributions from both the fine and coarse modes to the aerosol concentrations can be seen. Local wind and temperature characteristics, along with rainfall totals and relative humidity, were provided by the UK MET office from a collocated monitoring station.

Sampled INPs were extracted from filters by placing the filters into a centrifuge tube containing 5 mL Milli-Q® purified water, which was subsequently placed in a rotary mixer at 30 rpm. Detection of INPs was carried out using the Leeds microliter nucleation by immersed particles instrument (μL-NIPI)^65^. Nucleation was taken as the first visible occurrence of ice formation within the droplets, which was recorded using a high definition webcam. The cumulative number of INPs per mL of washing water (*K*(*T*)), was calculated using the singular approximation for ice nucleation as per Vali 1971^66^:

1 $$K(T)=\frac{-\mathrm{ln}(1-F(T))}{{V}_{d}}$$

where *F*(*T*) is the cumulative fraction of droplets frozen, and *V*_*d*_ is the droplet volume (0.001 mL). This can then be related to the number of INPs per litre of sampled air by:

2 $$[INP]=K(T)\times \frac{{V}_{ww}}{{V}_{air}}$$

where *V*_ww_ is the volume in mL of water used to wash the filter, and *V*_air_ is the volume of air sampled through the filter in L. For the heat treatment tests, 0.75 mL of the filter washing waters was placed into a sealed Eppendorf tube, and heated to 95 °C for 1 hour. Mass loss from heating was determined gravimetrically and accounted for. Following heating, particles were resuspended in the Eppendorf tubes using a vortex mixer for 20 s prior to analysis via the μL-NIPI. Daily control freezing spectra were taken of the ‘pure’ MilliQ® water, to ensure that any background freezing on that day (due to impurity particles in the water) was diminishingly small in number relative to the number of INPs in the water after aerosol sampling. The pure water backgrounds are illustrated in Supplementary Information Fig. 1.

A principle source of uncertainty in the experiments stems from the representativeness of sampling of INPs into droplets from the bulk suspension. This is of particular importance at temperatures where only a minor fraction of droplets contains active ice-nucleating particles. At such temperatures, the observed number of INPs per droplet in the subset sample removed from the vial can vary from that in the population (i.e. the vial of water into which INPs were sampled). Confidence intervals for the population number of INPs per droplet are constructed using the variance scores interval from Barker^67^:

3 $$\mu (T)+\frac{{({Z}_{\alpha /2})}^{2}}{2n}\pm {Z}_{\frac{\alpha }{2}}{[4\mu +{({Z}_{\frac{\alpha }{2}})}^{2}/n]}^{0.5}/{(4n)}^{0.5}$$

where *µ(T)* is the number of INPs per droplet, Z_α/2_ is the standard score at a confidence level *α*/2, which for a 95% confidence interval is 1.96.

### Global aerosol model

The GLOMAP mode global aerosol model used here has been described in detail in Mann *et al*.^68^. The horizontal resolution of the model is 2.8° × 2.8°, and contains 31 pressure levels from the surface to 10 hPa. The surface level is about 60 m deep. The model uses wind, temperature and humidity fields from the European Centre for Medium-Range Weather Forecasts (ECMWF) from the year 2000 to 2001 in order to reach a steady-state aerosol distribution before running the model for the year 2001 to 2002. Further details of the model setup are given in Vergara-Temprado *et al*.^6^, but the major uncertainties in the model arise from uncertainties in the evolution of dust concentrations and aerosol microphysical processes^69,70^, along with the differences in the density of active sites for different types of K-feldspar^71^ and the effects of acid aging on the feldspar nucleating activity.

### Back trajectory analysis

To examine the origin of air masses arriving at the rural site, 120 hr (5 day) air mass back trajectories arriving at 100 m were calculated using the NOAA HYSPLIT model v4^58^ using data from the Global Data Assimilation System archive. Hourly back trajectories were calculated to coincide with the sampling times. In order to probe whether the 5 day back trajectories could account for INP observations at the site, each trajectory was assigned a value of Easterly, Westerly, Northerly or Southerly, depending on the major origin of the air mass. As indicated in Fig. 3, INP levels were not found to be strongly influenced by the major direction of the back trajectory.

### Scanning electron microscopy and energy-dispersive X-ray spectroscopy

Details of the SEM-EDS analysis are provided in the supplementary information.

## Electronic supplementary material

Supplementary information

## Data Availability

The datasets generated during and/or analysed during the current study are available in the University of Leeds repository at, 10.5518/330.
