# Supplementary material for: Contributions of biogenic material to the atmospheric ice-nucleating particle population in North Western Europe
Source: Sci Rep. 2018 Sep 14;8:13821. doi: 10.1038/s41598-018-31981-7 (PMC6138667; doi:10.1038/s41598-018-31981-7)
Supplement: Supplementary file 1 — Supplementary information [file 41598_2018_31981_MOESM1_ESM.docx]

Supplementary Information for:

Contributions of biogenic material to the atmospheric ice-nucleating particle population in North Western Europe

D. O’Sullivan,^1^ M.P. Adams,^1^ M.D. Tarn,^1^ A.D. Harrison,^1^ J. Vergara-Temprado,^1^ G.C.E. Porter,^1^ M.A. Holden,^1,2^ A. Sanchez-Marroquin,^1^ F. Carotenuto^3^, T.F. Whale^1^, J.B. McQuaid^1^, R. Walshaw,^4^ D.H.P. Hedges,^4^ I.T. Burke,^5^ Z. Cui^1^ and B.J. Murray^1^

^1^ Institute for Climate and Atmospheric Science, School of Earth and Environment, University of Leeds, Woodhouse Lane, Leeds, LS2 9JT, UK

^2^ School of Chemistry, University of Leeds, Woodhouse Lane, Leeds, LS2 9JT, UK

^3^ Institute of Biometeorology, National Research Council (IBIMET-CNR), Via Caproni 8, 50145 Florence, Italy

^4^School of Earth and Environment, University of Leeds, Woodhouse Lane, Leeds, LS2 9JT, UK

^5^Earth Surface Science Institute, School of Earth and Environment, University of Leeds, Woodhouse Lane, Leeds, LS2 9JT, UK

Corresponding Author: Benjamin Murray Email: b.j.murray@leeds.ac.uk Tel: +44 (0)113 343 5605. Institute for Climate and Atmospheric Science, School of Earth and Environment, University of Leeds, Woodhouse Lane, Leeds, LS2 9JT, UK

**Supplementary tables and figures**

|  | Total number of runs = 51 | |
| --- | --- | --- |
|  | **Run Time (min)** | **Volume air sampled (L)** |
| Average | 178 | 888 |
| Max | 433 | 2165 |
| Min | 60 | 300 |

SI Table 1: Sampling times and volume of air sampled for the 51 runs performed during the campaign.

| Start | End | INP_-25°C_ | INP_-20°C_ | INP_-15°C_ | Runtime | Vol. air sampled |
| --- | --- | --- | --- | --- | --- | --- |
|  |  |  | *#/ L* |  | *(min)* | *(L)* |
|  |  |  |  |  |  |  |
|  |  |  |  |  |  |  |
| 26/09/2016 12:00 | 26/09/2016 15:00 |  | 0.6 |  | 180 | 900 |
| 26/09/2016 12:11 | 26/09/2016 15:12 |  | 0.4 |  | 181 | 905 |
| 27/09/2016 10:57 | 27/09/2016 14:26 |  | 0.6 | 0.2 | 209 | 1045 |
| 28/09/2016 12:35 | 28/09/2016 15:35 |  | 0.5 |  | 180 | 900 |
| 28/09/2016 13:34 | 28/09/2016 15:40 |  |  | 3.3 | 126 | 630 |
| 30/09/2016 10:19 | 30/09/2016 13:19 |  | 0.7 |  | 180 | 900 |
| 30/09/2016 11:20 | 30/09/2016 13:55 |  | 0.8 |  | 155 | 775 |
| 30/09/2016 14:21 | 30/09/2016 16:21 |  | 1.0 |  | 120 | 600 |
| 03/10/2016 10:20 | 03/10/2016 13:59 | 8.4 |  |  | 219 | 1095 |
| 03/10/2016 10:26 | 03/10/2016 13:27 | 7.9 |  |  | 181 | 905 |
| 03/10/2016 10:32 | 03/10/2016 13:35 |  | 0.5 | 0.2 | 183 | 915 |
| 03/10/2016 13:35 | 03/10/2016 16:36 |  | 1.8 |  | 181 | 905 |
| 03/10/2016 13:47 | 03/10/2016 16:23 |  |  | 1.2 | 156 | 780 |
| 04/10/2016 10:33 | 04/10/2016 13:20 | 12.3 | 2.8 |  | 167 | 835 |
| 05/10/2016 11:16 | 05/10/2016 12:18 | 17.7 |  |  | 62 | 310 |
| 05/10/2016 11:16 | 05/10/2016 15:59 |  | 11.4 |  | 283 | 1415 |
| 05/10/2016 13:39 | 05/10/2016 14:39 |  | 11.1 |  | 60 | 300 |
| 06/10/2016 11:39 | 06/10/2016 14:13 |  | 1.3 |  | 154 | 770 |
| 06/10/2016 11:40 | 06/10/2016 14:10 | 3.4 | 0.2 |  | 150 | 750 |
| 06/10/2016 14:20 | 06/10/2016 16:58 |  | 3.9 |  | 158 | 790 |
| 06/10/2016 14:24 | 06/10/2016 16:54 |  | 4.0 |  | 150 | 750 |
| 07/10/2016 10:41 | 07/10/2016 12:41 |  | 1.6 |  | 120 | 600 |
| 07/10/2016 11:21 | 07/10/2016 13:06 |  |  | 0.4 | 105 | 525 |
| 07/10/2016 13:02 | 07/10/2016 15:02 | 14.2 | 0.8 |  | 120 | 600 |
| 10/10/2016 11:29 | 10/10/2016 16:36 |  | 0.5 |  | 307 | 1535 |
| 10/10/2016 12:39 | 10/10/2016 13:39 |  | 1.9 |  | 60 | 300 |
| 10/10/2016 13:50 | 10/10/2016 16:36 |  | 0.4 |  | 166 | 830 |
| 11/10/2016 10:40 | 11/10/2016 14:03 | 10.0 | 0.6 |  | 203 | 1015 |
| 11/10/2016 14:10 | 11/10/2016 16:24 | 16.3 | 8.4 |  | 134 | 670 |
| 13/10/2016 10:39 | 13/10/2016 14:42 |  | 1.3 |  | 243 | 1215 |
| 13/10/2016 10:41 | 13/10/2016 14:42 |  | 0.9 |  | 241 | 1205 |
| 14/10/2016 07:30 | 14/10/2016 10:31 |  |  | 1.2 | 181 | 905 |
| 14/10/2016 10:39 | 14/10/2016 12:19 |  | 3.9 | 0.7 | 100 | 500 |
| 17/10/2016 11:46 | 17/10/2016 14:49 |  | 1.1 |  | 183 | 915 |
| 17/10/2016 15:10 | 17/10/2016 17:11 | 25.1 | 5.8 |  | 121 | 605 |
| 18/10/2016 10:45 | 18/10/2016 13:52 |  | 2.1 | 0.2 | 187 | 935 |
| 18/10/2016 10:55 | 18/10/2016 14:15 | 12.6 | 0.4 |  | 200 | 1000 |
| 18/10/2016 10:55 | 18/10/2016 16:10 |  | 0.3 |  | 315 | 1575 |
| 18/10/2016 14:00 | 18/10/2016 16:15 |  | 1.2 | 0.4 | 135 | 675 |
| 18/10/2016 14:15 | 18/10/2016 16:10 | 18.5 | 2.7 |  | 115 | 575 |
| 19/10/2016 10:04 | 19/10/2016 13:04 |  | 21.7 | 0.9 | 180 | 900 |
| 19/10/2016 13:20 | 19/10/2016 16:21 |  | 1.1 | 0.3 | 181 | 905 |
| 20/10/2016 11:45 | 20/10/2016 13:35 | 8.8 |  |  | 110 | 550 |
| 20/10/2016 13:45 | 20/10/2016 16:00 | 8.6 |  |  | 135 | 675 |
| 21/10/2016 11:42 | 21/10/2016 16:36 |  | 2.6 | 2.0 | 294 | 1470 |
| 21/10/2016 14:42 | 21/10/2016 16:36 |  | 4.1 | 3.9 | 114 | 570 |
| 24/10/2016 12:05 | 24/10/2016 15:37 |  | 1.6 |  | 212 | 1060 |
| 27/10/2016 11:49 | 27/10/2016 15:58 | 13.7 | 3.8 | 1.3 | 249 | 1245 |
| 31/10/2016 10:17 | 31/10/2016 17:30 | 6.8 | 0.4 |  | 433 | 2165 |
| 01/11/2016 11:10 | 01/11/2016 14:10 | 3.2 |  |  | 180 | 900 |
| 02/11/2016 10:29 | 02/11/2016 15:29 |  | 0.2 |  | 300 | 1500 |

SI Table 2: Extended data for the sampling periods


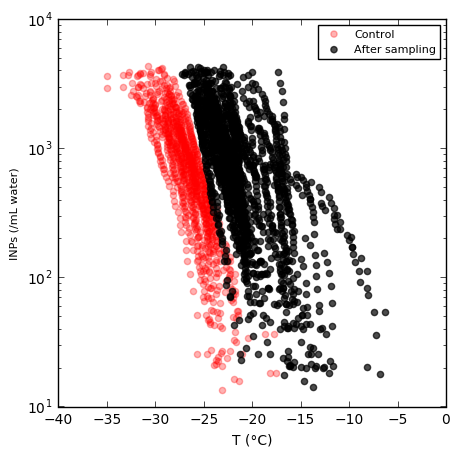


SI Fig 1: Data indicating the levels of INPs before and after sampling in the Milli-Q water. On any given day, INPs in the background were typically an order of magnitude less than those after sampling.


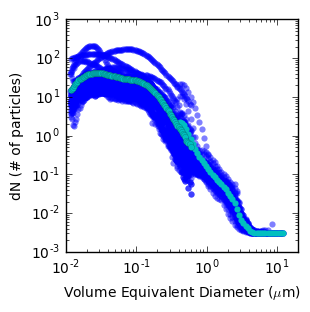


SI Fig 2: Compilation of aerosol particle size distributions measured throughout the campaign. The merged APS and SMPS measurements are shown in dark blue, while the average of all of these is shown in cyan.


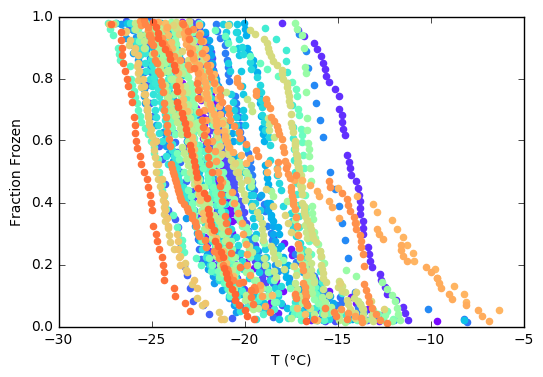


SI Fig 3: Compilation of all the experimental fraction frozen curves for the samples collected during the campaign.


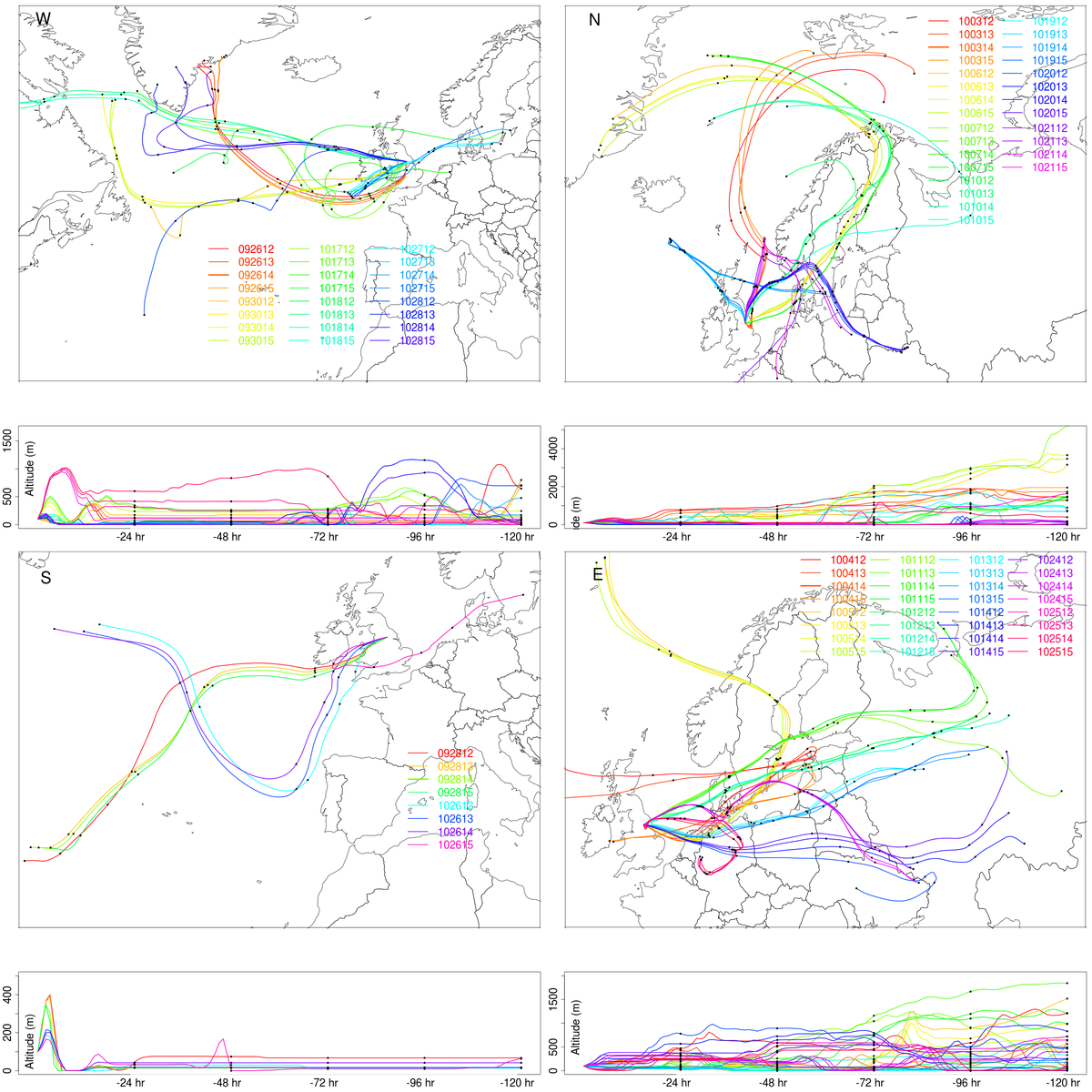


SI Fig 4: HYSPLITT back trajectory profiles. The letter in the top left corner indicates the assigned predominant direction for the trajectory which was used for the analysis in Fig 3 of the main paper. The trajectories are taken every hour during the sampling periods, and the altitudes of the back trajectories are in the panels beneath the main graph.

**SEM- EDS analysis**

Scanning electron microscopy was used to directly analyse the morphology and composition of the aerosol particles on one of the filters collected during the campaign (17^th^ October 2016). A similar approach has been used in Chou et al.,^1^ Hand et al.^2^ and Young et al.^3^ The polycarbonate filter was analysed using a Tescan VEGA3 XM scanning electron microscope (SEM) fitted with an X-max 150 SDD energy-dispersive X-ray spectroscopy (EDS) system controlled by AZtec 3.3 software. Prior to analysis, the filter was coated with iridium (30 nm) in order to make the filter substrate conductive. The SEM was operated at 20 keV, using a beam spot size that was adjusted to provide the optimum number of input counts for the EDS detector and a working distance of around 15 mm, with a Secondary Electron detector. Particle size, shape and compositional data was collected using the “AZtecFeature” software, optimised for particle analysis, as described below. Randomly chosen areas on the filter were scanned (avoiding being close to the filter edges) at two different magnifications, with a dwell time of 10 μs and a resolution of 1024 x 960 pixels per image. High magnification images (normally x5000) were used to detect particles down to 0.2 μm and medium magnification images (normally x1500) were used to scan particles down to 1 μm. Particles were identified based on their relative brightness compared with the background filter. The brightness threshold was determined manually for each area, in order to detect most of the small particles and minimize artefacts (primarily bright spots present at the edges of filter pores). The AZtecFeature software calculates the size and shape of each particle. Data was then expressed as the equivalent circular diameter of each particle which is defined as √(4*A*/π), where *A* is the cross sectional area of the particle.

EDS spectra were collected from the centre of some of the first particles detected in each area. The number of counts obtained per particle was around 50,000. From the X-ray spectrum of each particle, elemental weight percentages were calculated by the AZtecFeature software, and then used to categorise the particles into different compositional bins. Because of the fact that the interaction volume (~2-5 μm^3^) is in most cases larger than the particle volume, the X-ray spectra of each particle also contains peaks due to X-rays emanating from the polycarbonate filter, which is made of carbon and oxygen. As a consequence, the elemental weight percentages of the elements present in the particle would not correspond to the weight percentages obtained by the EDS analysis. Therefore, when categorising particles based on their composition, only the presence or absence of elements and the ratio between different elements was taken into account (see full scheme in SI Table 3). The compositionally categorised particles were then presented as the fraction of particles present in each category bin at each size bin (see SI Fig 5). In addition, we manually searched the filter for particles which were of obvious biological origin. These particles are shown in SI Fig 6 and are most likely fungal spores.


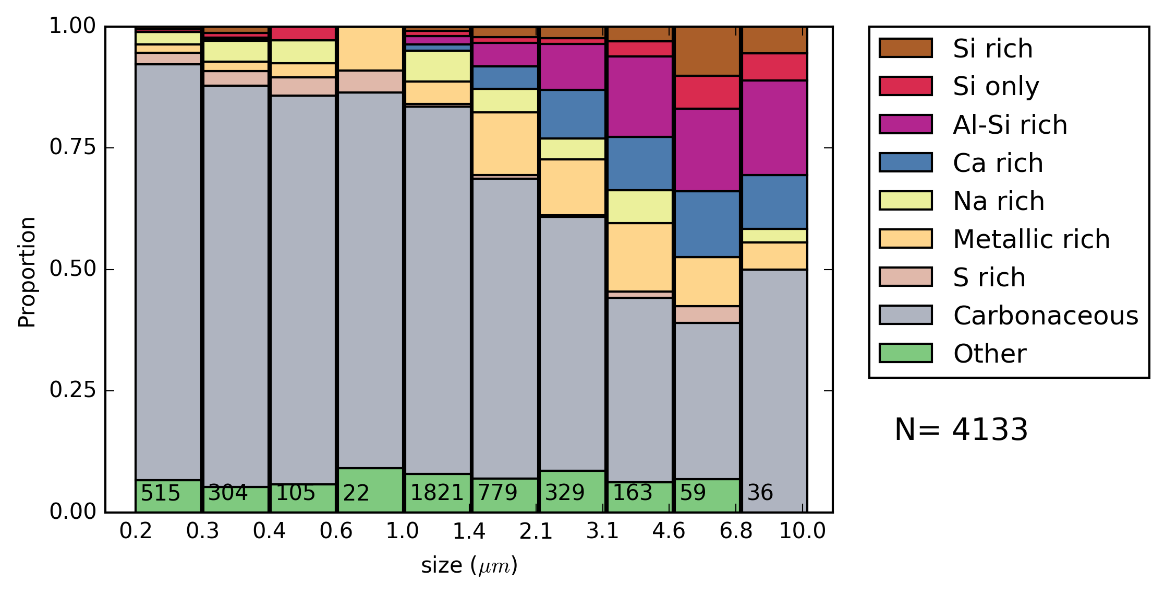


SI Fig 5: Size-resolved SEM-EDS analysis of a filter collected on 17^th^ October 2016, showing the fraction of particles belonging to each compositional category for each size bin according to their EDS spectra and equivalent circular diameter. A description and interpretation of the bins appears in SI Table 3. The most abundant group is carbonaceous particles, consistent with organic and black carbon particles. There is a significant contribution from Na and Cl rich particles (likely as NaCl) and metallic aerosol particles. The vast majority of particles in the categories Al-Si rich, Si only and Si rich are consistent with mineral dust. By summing these bins together, a mode of mineral dust can be seen in the supermicron size. The number of particles per bin is shown in the bottom of the bar. The total number of particles is also shown (N).


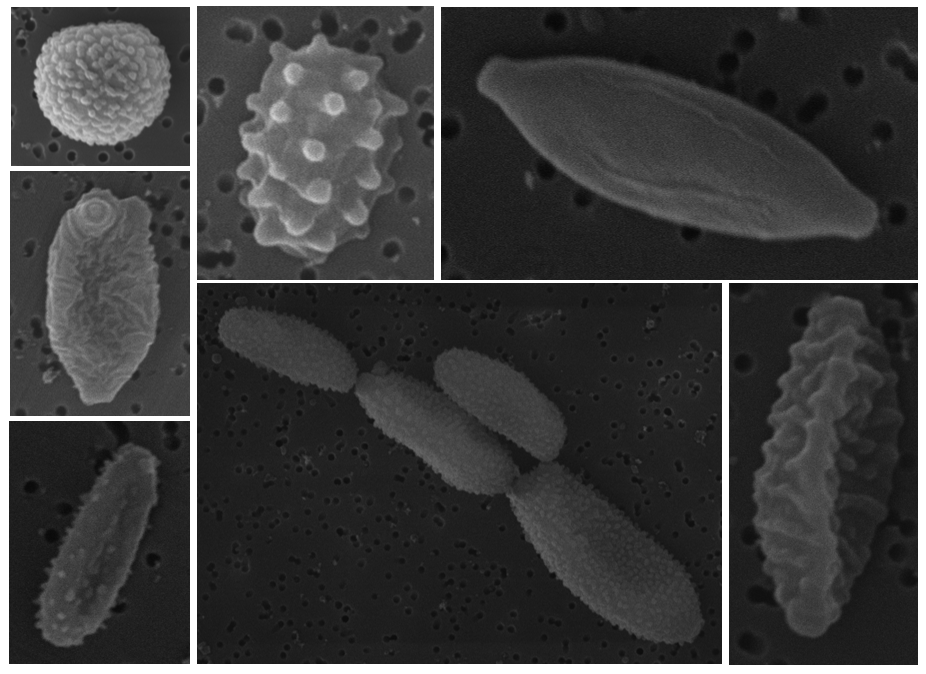


SI Fig 6: Scanning electron microscope images of a selection of particles which were obviously biological. These particles appear to be various types of fungal spore. The images demonstrate the presence of biological particles at the sampling site, but the statistics were poor and hence it was not possible to quantify their concentration. In addition, we were only able to identify biological particles by visual inspection, hence we most likely miss biological fragments and other biogenic-containing aerosol particle types. These particles fall into the carbonaceous category in SI Fig 5. For scale, the small black pores in the filter in the background of each image are 0.4 µm diameter.

| **Category** | **Primary** | **Secondary** | **Interpretation** |
| --- | --- | --- | --- |
| Carbonaceous | C+O | C+O  C+O+K  C+O+P | - black carbon, organic, biogenic |
| S rich | S |  | - sulphates |
| Metal rich | Fe/Al/Cr/Zn/Ti |  | - metal oxides,  metal rich aerosol |
| Na rich | Na | Na+Cl  Na+Cl+(Mn, Mg)  Na+Cl+S | - sea salt, aged sea salt |
| Al-Si rich | Al/Si | Al+Si  Al+Si+(Na, Mg, K, Ca, Fe, S) | - aluminosilicates |
| Ca rich | Ca | Ca  Ca+S  Ca+(Al, Si, Na, Mg, K, Ca, Fe) | - calcium carbonate, gypsum, Ca-rich mixed particles |
| Si only | Si |  | - silica |
| Si rich | Si | Si+(Na, Mg ,K, Ca, Fe) | - internally mixed silica, silicates |
| Other | Mixture | Mixture | - internally mixed particles, particles where the categorisation failed |

SI Table 3: Primary and secondary elements present each category and interpretation of the most likely type of aerosol. Secondary elements in parenthesis mean that at least one of the listed elements in the parenthesis was detected. C and O were present in the EDS spectra of all particles, so it is not mentioned after the first category. Due to the spatial resolution of the EDS, C and O were identified in each case from the polycarbonate filter, but may also have been present in the other aerosol particle types as black carbon, biogenic species, and as a component of organic and inorganic molecules (e.g. CaCO_3_).

**References**

1. Chou, C. *et al.* Size distribution, shape, and composition of mineral dust aerosols collected during the African Monsoon Multidisciplinary Analysis Special Observation Period 0: Dust and Biomass-Burning Experiment field campaign in Niger, January 2006. *J. Geophys. Res. Atmos.* **113**, D00C10 (2008).

2. Hand, V. L. *et al.* Evidence of internal mixing of African dust and biomass burning particles by individual particle analysis using electron beam techniques. *J. Geophys. Res. Atmos.* **115**, D13301 (2010).

3. Young, G. *et al.* Size-segregated compositional analysis of aerosol particles collected in the European Arctic during the ACCACIA campaign. *Atmos. Chem. Phys.* **16**, 4063–4079 (2016).
